# Supplementary material for: Analyzing lumbar vertebral shape and alignment in female patients with degenerative spondylolisthesis: Comparisons with spinal stenosis and risk factor exploration
Source: PLoS One. 2024 Apr 16;19(4):e0301974. doi: 10.1371/journal.pone.0301974 (PMC11020370; doi:10.1371/journal.pone.0301974)
Supplement: S1 Checklist — (DOCX) [file pone.0301974.s001.docx]

**Human Participants Research Checklist**

***Complete the following if your study involved human participants or human participants’ data. These questions should be addressed for prospective and retrospective studies.***

3.If you are reporting a retrospective study of medical records or archived samples, please report in the Methods section:

1. the day, month and year when the data were accessed for research purposes

→ 02/20/2022

1. whether authors had access to information that could identify individual participants during or after data collection

→Accessed personal information during data collection
